# Supplementary material for: Learning curve and surgical outcome of robotic assisted colorectal surgery with ERAS program
Source: Sci Rep. 2022 Nov 29;12:20566. doi: 10.1038/s41598-022-24665-w (PMC9709162; doi:10.1038/s41598-022-24665-w)
Supplement: Supplementary file 1 — Supplementary Information 1. [file 41598_2022_24665_MOESM1_ESM.zip › LSC Raw data-Ñ[▒K/123/H_ñw╝╨░O▒Kñσ.pdf]

正式摘要(電子簽章：0000021557 5843)

W95 / W95 ☐英文標題(注意:無英文姓名-請補病人基本檔!) ☐【讀取】憑證

臺中榮民總醫院

出院病歷摘要

轉入號： 轉出號：

[redacted]

(8)流水編號	
(9)入院日期	110/03/15 (2021) 直腸外科 W95 - 02
(10)轉科日期	
(11)出院日期	110/04/26 (2021) 住院天數計 42 日

診斷

- 入院:Rectal tumor
- 出院:1. Rectal cancer with total obstruction
  - s/p loop S colonostomy on 2021/03/15
  - s/p laparoscopic converting open low anterior resection + loop ileostomy formation on 2021/03/23
  pT3N1a(1/12)M0, pMMR, MD, AI+, PI+, TuD+, CRM-, stage IIIA
  2. COPD and KP pneumonia s/p ETT s/p med.

主訴

- Acute abdominal pain with nausea/vomitting at 8PM tonight.

病史

- The 85 year-old man had history of 1. s/p appendectomy 2.right patella fracture s/p ORIF, BPH s/p TURP*2
  This time, he suffered from acute abdominal pain with nausea/vomitting at 8PM 3/15, then he was brought to our ER for help. There was no diarrhea, no fever but coldness, no back pain, no chest pain. Abdominal CT showed irregular wall thickness noted at proximal rectum, length about 4-5 cm. R/O rectal ca. Dilatation of colon from ascending colon to rectum, with much fluid retention. Bowel obstruction was highly suspected. New diagnosed rectal cancer with bowel obstruction was suspected. Flomoxef was prescribed empirically. CRS was consult and Loop colostomy was done on 2021/03/15. He was admitted for post op care.

體檢發現

- Fundoscopy:
  VFT:
  - General appearance :
    - acute ill looking , conscious clear , without cardiopulmonary distress
    - Vital signs自動帶入date 體溫(BT) 脈搏(P) 呼吸(R) 血壓(BP) 身高(BH) 體重(BW) BMI
      20210315 37.4C 72/min 18/min 135/77mmHg 176cm 65kg 21.0
    - Vital signs: B.P. 135/77 mmHg , P.R. 72 /min , R.R. 18 /min , B.T. 37.4 ℃
  - HEENT :
    - Eyes: no anemic conjunctiva, no icteric sclera, normal EOM
    - ENT: no otorrhea, no rhinorrhea, no erythematous mucosa, no injected tonsil, no deformity
  - Neck: supple, freely movable, trachea midline,no deformity, no engorged jugular vein , no palpable thyroid or lymph node enlargement
  - Chest:
    - Inspection: Grossly normal, symmetric and fully expansion, no accessory muscle used, no operation scar, no spider angiomata, no gynecomastia
    - Palpation: no palpable mass or nodule, no subcutaneous emphysema, no tenderness
    - Ausculation: clear breathing sound
  - Heart:
    - Percussion: normal heart size
    - Ausculation: regular heart beat, normal S1, S2, no gallop, no murmur
  - Abdomen:
    - Inspection: in
      - flat contour, symetrically, no scar, no stria, no dilated veins, no rashes
    - Ausculation: normoactive bowel sound, no abdominal bruits

-
    - palpation: soft, no muscle guarding or rigidity, no local tenderness, no rebounding pain, no palpable mass, no palpable pulsation
    - Percussion: no shifting dulllness, no hepatomegaly, no splenomagaly
-

Peripheral pulsation :	CA	BA	RA	FA	PA	PTA	DPA
Right side :	++	++	++	++	++	++	++/+++
Left side :	++	++	++	++	++	++	++/+++

- Genito-anus: no tenderness or palpable mass lesion or rectal shelf, normal external genitalia, normal anal tone, brown stool

CRS CONDITION

- Imaging studies(Sono , CT of abdomen and chest Films):
    - Irregular wall thickness noted at proximal rectum, length about 4-5 cm. R/O rectal ca.
      > Multiple small diverticulosis noted at sigmoid colon, with surroudning mild rection. R/O diverticulitis.
      > Dilatation of colon from ascending colon to rectum, with much fluid retention. Bowel obstruction was highly suspected.
      > Multiple lymphadenopathy at left hemipelvis upt o 1.7 cm. Metastasis lymphadenopathy can not be completely excluded.
-
    - Lung

手術日期及方法

- 手術日期及方法20210315 Loop colostomy Colon, Colonofibroscopy with polypectomy.
  20210324 Low anterior resection +/- pelvic LN dissection Ileostomy Laparoscopy

住院治療經過

- The patient was admitted for low anterior resection with colo-anal anastomosis. After admission, new diagnosed rectal cancer with bowel obstruction was suspected according to abdominal CT. Flomoxef was prescribed empirically. Loop colostomy was done on 2021/03/15. Tumor staging, lung CT, plevis MRI and PET were arranged and done. Laparoscopic Anterior resection was performed on 3/24. Try water on 3/27, and start try Bland -I diet on 3/28. Fever episode was noted on 3/28, U/C,S/C were collected and keep current antibiotic use. We added albumin mixed lasix for CXR bil. pleural effusion. We suspected chyle leakage from penrose and central wound on 3/30, even TG: 126mg/dL (unlikely). NG feeding with low fat since 3/31. U/C: Pseudomonas aeruginosa, S/C: Klebsiella pneumoniae was found and after infection consulting, Tazocin was prescribed. We switched antibiotics to Cefepime according to INF suggestion. On 4/2, he experience a choking episode with symptoms of dyspnea and being desaturated. Blood gas showed respiratory acidosis with severe CO2 retension. CXR revealed bilateral lung infiltration. We soon initiated O2 therapy with BiPAP. His repiratory symptoms alleviated after treatment. He is currently under intermittent BiPAP use and we would follow-up this condition. His symptoms of chyle leakage also improved. He started NG feeding after condition became stable. On 4/6, he became feverish and we soon arranged fever survey including blood culture. His respiratory status was rather smooth. We keep current antibiotics regimen and close surveillance on his condition. However, his respiratory status became worse on 4/7 night and was therefore intubated and then transfered to SICU. At SICU, inhalation agent was added according to Doctor Fu. The patient start to try weaning on 4/10. The respirator pattern was smooth while trying weaning. The weaning index was checked and endotracheal tube was removed on 4/12. Under stable condition, he was transferred to ordinary ward. At ordinary ward, he begane rehabilitation program to improve mobility. His could walk with the aid of walker. Under relatively stable condition, he was discharged on 2021/4/26 with OPD follow-up.

合併症

一般檢查

- 累積報告 :

DATE	TIME	ALB	TP	BIL.T	BIL.D	ALKP	AST	ALT	LDH	CHOL	TG	HDL-C	LDL	UA	NH3	AMY
1100314	2354			0.5				13								
1100317	0852	2.4	4.8	0.5	0.1		25	6								
1100322	1421	2.4		0.32	0.11		40	6								
1100324	1640	2.7		0.46	0.16	68	25	9								
1100328	2020			0.54	0.21		17	4								
1100330	1621										126					
1100331	2222	3.2		0.65	0.28		13	3								
1100402	1814	2.9														
1100402	2320														65	
1100406	1948	3.4		0.65	0.25		108	39								
1100407	2145	3.6		0.64	0.24		97	58								
1100408	0005			0.6	0.2											
1100408	0525			0.51	0.20	179	55	45								
1100409	0444			0.27	0.14		128	66								
1100410	0512			0.3	0.1		91	72	173							
1100414	0614			0.2	0.1											
1100414	1540						43	38								

1100419	0903	3.4					64	41								

DATE	TIME	WBC	RBC	HGB	HCT	MCV	PLT	BLST	NEUT	BAND	SEG	LYM	MON	EOS	BAS
1100314	2354	21850		13.1			254			3.0	91.0	4.0	2.0	0.0	0.0
1100317	0907	12600	3.46	10.7	32.4	93.6	186		86.7			7.0	6.0	0.1	0.2
1100322	1412	6080	3.82	11.5			270		75.1			10.2	9.4	4.8	0.5
1100324	1640	13750		9.5			245		94.2			3.7	1.8	0.2	0.1
1100328	2019	11650		10.0			343		91.7			2.0	5.8	0.3	0.2
1100331	2222	9180	3.02	9.0			254		85.2			5.2	8.7	0.8	0.1
1100402	1813	17300	3.08	9.1	30.0	97.4	281		88.0			4.0	7.3	0.5	0.2
1100406	1935	8470	2.85	8.5			305		80.1			6.5	9.7	3.5	0.2
1100407	2137	13150	3.50	10.4	32.5	92.9	339		92.8			5.6	1.2	0.2	0.2
1100408	0004	10900	3.33	9.9	31.2	93.7	331		96.6			2.3	1.0	0.0	0.1
1100408	0522	9850		9.2			303		91.6			4.0	4.1	0.1	0.2
1100409	0443	10100		9.4			317		85.2			4.6	7.0	3.0	0.2
1100410	0509	8480	3.16	9.4	29.3	92.7	309		78.5			7.4	9.3	4.6	0.2
1100412	0458	9270		9.1	28.8		299		78.1			10.1	7.2	4.3	0.3
1100414	0544	8420	3.27	9.6	29.8	91.1	366		80.9			10.9	6.7	1.1	0.4
1100419	1004	5670	3.58	10.8	32.1	89.7	343		57.9			26.5	8.6	6.3	0.7

DATE	TIME	NA	K	CL	CA	BUN	CREAT	Mg	P	CRP	ESR	PCT	LIPASE	LACTATE
1100314	2354	137	4.0			24	0.84			0.308			24	33.8
1100315	0538													34.2
1100317	0852	138	3.8		7.4	22	0.90							
1100322	1421	139	3.4	105	7.3	8	0.84							
1100324	1640	140	3.6	104	7.4	5	0.80							
1100328	2020	140	3.7		7.2	22	0.84							12.8
1100331	2222	142	2.6	97	7.8	25	0.83							
1100402	1814	140	2.6			24	0.79							17.2
1100402	2104							1.5	1.1					
1100402	2120											1.610		
1100403	0903	143	3.5			28	0.97							
1100406	1947													10.7
1100406	1948	138	3.9	106	8.3	31	0.81			4.857				
1100407	2145	135	5.5	105	8.6	31	0.85	2.3	3.0					
1100407	2146													11.0
1100408	0005	137	5.3				0.91							
1100408	0524													11.1
1100408	0525	141	4.8	109	8.8	36	0.90							
1100408	0527											1.1		
1100408	1550		4.2											
1100409	0444	140	4.6			41	0.94							
1100410	0509													6.9
1100410	0512	138	4.8	105	8.5	36	0.99							
1100411	0521		5.0											
1100412	0500	137	4.4		8.3	44	0.95							
1100413	0507		4.3											
1100414	0614	141	3.8				0.86							
1100414	1540					44								
1100419	0903	134	5.4			25	0.90							

DATE	TIME	GLUCOSE	PAB	TRF	ZN	CU	KETONE	CK	CKMB	PT-P	PT-C	APTT-P	APTT-C	TRO-T
1100314	2354	203						49		11.0	10.6	25.4		
1100315	0009													16.55
1100324	1640	232												
1100331	2222									15.2	10.6	40.7		
1100402	2320							8						32.13
1100402	2321								3					
1100403	0857						0.0							
1100407	2145							10	3					
1100407	2146													36.19
1100408	0524													34.36
1100408	0525							8						
1100408	0526								3					

DATE	TIME	SP-GR	PH	PROT	GLUC	KETON	BILI	UROB	OB	NITR	LEU	RBC	WBC	EPCEL
1100315	0124	1.020	5.0	Trace	Negative	Trace	2+	2.0	Negative	Positive	Trace	0-2	0-2	0-1
1100329	0038	1.019	5.5	Trace	Negative	Negative	Negative	0.2	Negative	Negative	2+	0-2	20-35	1-2
1100402	2144	1.019	5.5	1+	>1000.0	Negative	Negative	0.2	Trace	Negative	1+	2-5	20-35	0-1
1100406	1739	1.015	5.0	1+	250.0	Negative	Negative	0.2	2+	Negative	1+	20-35	20-35	20-30
1100408	0013	1.014	5.0	1+	Negative	Negative	Negative	0.2	3+	Negative	1+	10-20	10-20	4-6

DATE	TIME	PH	PCO2	PO2	SO2	BEB	HCO3	TCO2	HGB	FIO2
1100402	2319	7.135	97.0	271.8	99.7	-0.5	31.9	34.9		

1100403	1800	7.404	47.4	190.1	99.7	3.4	28.9	30.4		
1100407	2136	7.350	42.1	66.5	91.6	-2.8	22.7	24.0		
1100407	2358	7.303	44.8	256.2	99.8	-4.7	21.7	23.1		
1100410	0512	7.429	43.4	205.7	99.7	3.3	28.1	29.4		
1100414	0553	7.480	41.6	84.6	97.3	6.2	30.3	31.6		

DATE	TIME	PH	BEB	SCVO2	PO2	HCO3	HGB	PCO2	TCO2
1100314	2355	7.285	0.3	29.6	22.0	28.5		61.3	30.4
1100315	0539	7.313	-1.7	63.3	36.5	25.2		50.7	26.7
1100324	1639	7.362	-0.7	98.4	118.5	25.1		45.1	26.4
1100402	1812	7.373	5.3	93.8	71.1	32.3		56.7	34.0

特殊檢查

病理報告

- 活體組織切片病理報告　手術日期：**110/03/24**　病理號：**S1009828**

診斷醫師:吳致瑩 報告日期:110/04/03
Pathologic diagnosis:
1. Rectum to sigmoid colon, laparoscopic anterior resection --- Adenocarcinoma, moderately differentiated. Tumor invaded to pericolorectal soft tissue with one tumor deposit and and metastasized to mesocolic lymph nodes (1/11). The bilateral cut ends are free of tumor.
2. IMA root, dissection --- Unremarkable fibroadipose tissue and lymph node (0/1).
3. Rectum, cut end, distal, resection --- Unremarkable.
Ancillary study for diagnosis:
1. IHC stains for PMS2 and MSH6 are done on section E. Both markers are preserved.
2. IHC stains for actin M851 (+) and HMB-45 (-) done for section K. Focal lymphatic channels proliferation found.
Prognostic and predictive factor:
1. Tumor size: 3x2.5 cm.
2. Depth of invasion: Pericolorectal tissue.
3. Histologic grade: G2: Moderately differentiated.
4. Angiolymphatic invasion: Present.
5. Perineural invasion: Present.
6. Tumor deposits (discontinuous extramural extension): Present, specify number of deposits: 1.
7. Macroscopic tumor perforation: Not identified.
8. Surgical Margins: a) Proximal Margin: Uninvolved by invasive carcinoma, distance of tumor from margin: 17 cm. b) Distal Margin: Uninvolved by invasive carcinoma, distance of tumor from margin: 2 cm. c) Radial or Mesenteric Margin: Uninvolved by invasive carcinoma, distance of tumor from margin: 1.5 cm.
9. Lymph node status: Involved (1/12).
10. Extranodal involvement: Not identified.
11. Tumor regression grading S/P CCRT: No known presurgical therapy.
12. Type of polyp in which invasive carcinoma arose: Not identified.
13. Additional pathologic findings: Not identified.
14. Pathological TNM Stage: pT3N1a(According to the eighth edition, American Joint Committee on Cancer Staging Guidelines for Tumors).
15. TNM descriptors: Not applicable.
Gross description:
The specimen consists of 1) a segment of colon, 20 cm in length and 4.8 cm in proximal cut end circumference, 5 cm in distal circumference, and mesocolic and perirectal soft tissue. A gray tan firm annular elevated and centrally ulcerative tumor, 3x2.5 cm, is noted, 2 cm to the distal cut end. On cross section, the tumor invades to the mesocolic soft tissue with 15 mm to the radial margin, and serosal retraction is also noted. There is focal mucosal ulceration with lumen narrowing noted 2 cm from the proximal cut end. There is exduate coating noted at the mesocolon near the narrowing site. The both cut ends are free of tumor grossly. 2) IMA root, a piece of tan yellow soft tissue, 4.5x2.5x1.8 cm. 3) distal cut end, a piece of tan mucosa-covered soft tissue, 1.8x1.5x0.9 cm.
Representative part for section: A) distal cut end B) proximal cut end C-F) tumor G) radial margin H-J) narrowing site K) specimen 2 L) specimen 3 M-P) lymph nodes. Blue ink indicated serosa; yellow ink indicated radial margin.

- Note:
  1. The interpretation of MMR panel and recommended studies are as below: (1) No loss of nuclear expression of MMR proteins: low probability of microsatellite instability-high (MSI-H) . (2) Loss of nuclear expression of MLH1 and PMS2: testing for methylation of the MLH1 promoter and/or mutation of BRAF is indicated (the presence of a BRAF V600E mutation and/or MLH1 methylation suggests that the tumor is sporadic and germline evaluation is probably not indicated; absence of both MLH1 methylation and of BRAF V600E mutation suggests the possibility of Lynch syndrome, and sequencing and/or large deletion/duplication testing of germline MLH1 may be indicated). (3) Loss of nuclear expression of MSH2 and MSH6: high probability of Lynch syndrome (sequencing and/or large deletion/duplication testing of germline MSH2 may be indicated, and, if negative, sequencing and/or large deletion/duplication testing of germline MSH6 may be indicated). (4) Loss of nuclear expression of MSH6 only: high probability of Lynch syndrome (sequencing and/or large deletion/duplication testing of germline MSH6 may be indicated). (5) Loss of nuclear expression of PMS2 only: high probability of Lynch syndrome (sequencing and/or large deletion/duplication testing of germline PMS2 may be indicated). (6) Please note: There are exceptions to the above IHC interpretations. These results should not be considered in isolation, and clinical correlation with genetic counseling is recommended to assess the need for germline testing.
  2. This case has been peer reviewed by two doctors.
  #T-59600_2 #M-81403_2 2 1105 S0000F
  #T-C4560_2 #M-81406_2 0 1105 000000

其它

出院時情況

- 3.改門診治療

出院指示

藥名	劑量	劑量單位	用法	頻次	數量	數量單位	現況	天數	單位
Nincort oral geL 6gm		TUB	TOP	TID	0	TUB	TH	1	瓶
Acetylcysteine tab	1	TAB	PO	BID	14	TAB	TH	7	天
Ultracet tab	0.5	TAB	PO	BID	7	TAB	TH	7	天
(口)Takepron OD tab 30mg	1	TAB	PO	QDAC	7	TAB	TH	7	天
BeroduaL 10mL inh	2	PUFF	INH	TID	1	PUFF	TH	7	天

出院計劃

- 門診日期
  - date on 2021/05/03

主治醫師 2222H 林俊余 / 住院醫師 9958C 林強生

正式摘要(電子簽章：000000215575843)

W95 / W95

doctorRank:B
